# Intravenous misuse of slow-release oral morphine capsules: how much morphine is injected?

**DOI:** 10.1186/s12954-023-00781-2

**Published:** 2023-04-27

**Authors:** Célian Bertin, Edouard Montigne, Sarah Teixeira, Florent Ferrer, Louis Lauwerie, Damien Richard, Nicolas Authier

**Affiliations:** 1 Université Clermont Auvergne, CHU Clermont-Ferrand, Inserm 1107 Neuro-Dol, Service de Pharmacologie Médicale, Centres Addictovigilance et Pharmacovigilance, Centre Evaluation et Traitement de la Douleur, Université Clermont Auvergne, 63003 Clermont-Ferrand, France; 2 Observatoire Français des Médicaments Antalgiques (OFMA)/French Monitoring Centre for Analgesic Drugs, CHU Clermont-Ferrand, Université Clermont Auvergne, 63001 Clermont-Ferrand, France; 3 UFR Médecine et Professions Paramédicales, Fondation Institut Analgesia, 63001 Clermont-Ferrand, France

**Keywords:** Person who inject drugs, Morphine, Opioid use disorder, Misuse, Harm reduction

## Abstract

**Background:**

The injection of morphine from morphine sulfate capsules containing sustained-release microbeads (Skenan®) is a practice frequently described by French intravenous opioid users. They seek an injectable form of substitution for heroin. Depending on how the syringe is prepared, the morphine rates may vary. The dosage of the capsule, the temperature of the dissolving water and the type of filter used have been identified as the parameters most likely to influence the final quantity of morphine in solution before intravenous injection. The aim of our study was to determine the amounts of morphine actually injected, according to the different preparation modalities described by people who inject morphine and the harm reduction equipment made available to them.

**Methods:**

Different morphine syringes were prepared by varying the dosage of the capsule (100 or 200 mg), the temperature of the dissolving water before adding morphine, ambient (≈ 22 °C) or heat (≈ 80 °C) and four filtration devices: risk reduction Steribox® cotton, risk reduction filter “Sterifilt®”, “Wheel” filter and cigarette filter. The quantification of the morphine in the syringe body was carried out by liquid phase chromatography coupled with a mass spectrometry detector.

**Results:**

The best extraction yields were obtained with heated water, independently of dosages (*p* < 0.01). Yields of 100 mg capsules varied according to the filter (*p* < 0.01) and the water temperature (*p* < 0.01), with maximum yields obtained for solutions dissolved in heated water, then filtered with the “Wheel” filter (83 mg). The yields of the 200 mg capsules varied according to the temperature of the water (*p* < 0.01), without difference according to the filter used (*p* > 0.01), and maximum yields obtained for solutions dissolved in heated water (95 mg).

**Conclusions:**

No procedure for dissolving Skenan® led to the complete dissolution of the morphine it contains. Whatever the variations in preparation conditions, the extraction rates of the 200 mg morphine capsules were lower than those of 100 mg, without the risk reduction filters adversely impacting morphine extraction. Offering an injectable substitution to persons who inject morphine would make it possible to reduce the risks and damage, particularly overdoses, associated with variations in dosage due to preparation methods.

**Supplementary Information:**

The online version contains supplementary material available at 10.1186/s12954-023-00781-2.

## Background

Although prescription opioid analgesic use disorder and its complications is a reality for the majority of industrialized countries [1], France remains globally spared by this phenomenon, thanks to its strict regulations on advertising by the pharmaceutical industry, the prescription and dispensing of opioid medications, most of which are classified as narcotics, and an effective addictovigilance system [2–6]. Despite the number of French people treated with opioid analgesics remaining stable [2], French addictovigilance systems have reported the existence of misuse of a particular form of sustained-release morphine sulfate, marketed under the name Skenan®, and at its highest doses (100 and 200 mg) [6–10].

This opioid analgesic medication is diverted from its indication by several thousand people suffering from opioid use disorder (OUD) either as an alternative or a complement to the only two validated oral opioid substitution treatments (OST—buprenorphine and methadone), or as a replacement for an illicit opioid, heroin [9–14]. Patients justify the use of morphine by intolerance to or the ineffectiveness of validated OST, the impossibility of doing without the intravenous route, increased ease of access, and better control of the quality and quantity of opioids administered compared to heroin [9, 10]. The diverted morphine is provided either by medical prescriptions, sometimes falsified or resulting from medical nomadism, delivered in pharmacies or by purchase on illicit markets [10, 15, 16].

People who use morphine in the context of OUD report that it is mostly administered intravenously, which implies the dissolution of the oral capsule of Skenan®, which is not consistent with the summary of product characteristics (chapter 4.2: “Administration”) [8, 9, 11]. To this end, the contents of Skenan® capsules are crushed, dissolved and filtered before being injected [17]. This practice is associated with increased risks of overdose, infectious complications, and thrombosis, by a defect of filtration of some oral excipients, compared to conventional OST [11, 12, 18–20]. Although intravenous administration is theoretically associated with 100% bioavailability, the real amount of morphine contained in these syringes and then injected is very little described [21].

According to the people who inject morphine intravenously (PWIM), the quantities of morphine in solution varied according to three main parameters: the dosage of the capsule, the temperature of the dissolving water and the type of filter used. Lack of knowledge of the administered dose and its potential variations can be a source of dosing error for PWIM. It can also lead to difficulty for the physician when adapting an OST prescription, and it can be a barrier for the experimentation of morphine-based injectable opioid substitution treatment for some people suffering from OUD for whom the use of the intravenous route constitutes a behavioral dependency in itself.

The aim of this study was to quantitatively assess the morphine dose in the syringe prepared for injection, in order to determine the actual amounts injected according to the different preparation conditions described by the PWIM.

## Methods

To facilitate the concordance of the results of this study with the practice of PWIM, the protocol used reproduced the preparation methods described on the main French open-access self-support and risk and harm reduction (RHR) forum “Psychoactif” [17].

### Material

The equipment used was identical to that made available to PWIM by the self-support and RHR associations (Additional file 1: Fig. S1). The Stericup® preparation cookers available in the Steribox® lower-risk injection kit (marketed by “Apothicom”), as did the 5 ml vials of water for injection. The 2 ml syringes were used, in line with the higher volume of water generally used by PWIM. In order to assess the impact of the filtration devices on the final amount of morphine found in the syringe, the four types of filters that appeared to be most common when the study was designed in 2020, were tested [22]: the “cotton” filter from the Steribox® kits, a filter made of cellulose acetate from industrial cigarettes, and two single-use commercial syringe filters consisting of a sterile filtering membrane “Sterifilt® Basic” (pore size: 10 µm) [23] and “Wheel” filter (diameter 25 mm, pore size: 0.22 µm) [24] (Additional file 1: Fig. S1).

The morphine-based medication, Skenan®, is a sustained-release capsule, marketed by the European pharmaceutical company “Ethypharm” for the treatment of intense persistent pains or those resistant to other analgesics, in particular of cancerous origin. The capsules contain morphine microbeads coated with Aquacoat® ECD 30, a gastro-resistant coating polymer which constitutes a hydrophilic matrix that gels on contact with water, allowing the prolonged release of the active ingredient [25]. The bioavailability of oral morphine compared with intravenous morphine is 30% [26]. Skenan® capsules were supplied by the hospital pharmacy of the University Hospital of Clermont-Ferrand in 100 and 200 mg doses, corresponding to those most frequently requested by users [9].

### Morphine sulfate dissolution process (Additional file 1: Fig. S2)

The capsule was opened to extract the morphine microbeads. These were placed in a paper pouch and crushed into a fine powder (Additional file 1: Fig. S2—steps 1–3) to facilitate the dissolution of the morphine. The powder was poured into a Stericup® (step 4), into which 2 ml of sterile water, at room temperature (≈ 22 °C) or heated (≈ 80 °C), was added. In the second case, the water was previously heated in an empty sterile cup, before being added to the powder (steps 5–7). According to PWIM, this method improves the water solubility of morphine, without causing the increase in viscosity described when directly heating the solution of water and Skenan® powder. The solution was mixed with the plunger of the syringe for about 40 s to homogenize the contents of the Stericup® and promote the dissolution of the morphine. The solution was then aspirated into the syringe through one of four filters (step 8 and 9). The “Wheel” filter required the preliminary humidification of its membrane by aspiration of 0.3 ml of sterile water in the syringe. This humidification water was then eliminated from the syringe, before aspiration of the morphine solution through the “Wheel” filter. In accordance with the recommended practice, the “Wheel” filter was rinsed after filtration by aspiration of 3 drops of sterile water, ensuring the aspiration of the morphine solution retained in the filter [22]. The filtered solution was then transferred to a hemolysis tube (step 10) and subjected to ultrasound for 5 min to homogenize the solution.

A set of 112 syringes was prepared by two technicians following the same handling protocol, corresponding to a series of seven syringes for each of the two dosages of Skenan®, repeated for each of the four types of filters, and each water temperature condition, heated or not.

### Analysis

The identification and quantification of dissolved morphine was performed by liquid chromatography coupled to a high-resolution mass spectrometry detector (Exactive®, Thermo Fisher Scientific®). Quantitative measurements were performed using a calibration curve in aqueous matrix, according to the technique of dosed additions of morphine. A specific internal calibration, by the addition of stable deuterated morphine (morphine-D3) in all the samples, guaranteed the accuracy of the analyses. All the samples were analyzed with the same analytical procedure.

The quantitative variations of morphine in the syringes according to the dosages of Skenan®, and the conditions of preparation and then filtration were compared using the statistical test of variance analysis (ANOVA) without and then with interaction. Tukey’s range test was performed to compare the means between them. All statistical analyses were performed using R Statistical Software (version 2.14.0; R Foundation for Statistical Computing, Vienna, Austria). The threshold for statistical significance was defined as *p* < 0.05.

## Results

### Skenan® 100 mg capsules

Univariate analysis without interaction showed a significant difference in the amounts of morphine released into solution according to variations in filter and water temperature [*F* = 6.1; *p* < 0.01] when dissolving the Skenan® 100 mg capsules.

The analysis with interactions found significant variations in extraction yields, as well as variations in temperature of the dissolution water (*p* < 0.01), likewise for the type of filters used (*p* < 0.01), although there was no interaction between these variations in the preparation conditions (*p* = 0.5). The average extraction yields were 66% (i.e., 66 mg) of morphine per syringe in heated water and 56% (i.e., 56 mg) of morphine per syringe in water at room temperature (Table 1).

**Table 1:** Average extraction rates of morphine in syringes, according to variations in preparation conditions

	Water temperature	Number of syringes per filter	Steribox® filter		Cigarette filter		Sterifilt® basic filter		“Wheel” filter		Average rates	
			Recovery Rate (%)	Dosage (mg)	Recovery rate (%)	Dosage (mg)	Recovery rate (%)	Dosage (mg)	Recovery rate (%)	Dosage (mg)	Recovery rate (%)	Dosage (mg)
Skenan® dosage												
100 mg	Heated (80 °C)	7	68.05%	68.05 mg	58.10%	58.10 mg	54.75%	54.75 mg	82.60%	82.60 mg	65.88%	65.88 mg
			SD = 13.17%	SD = 13.17 mg	SD = 10.92%	SD = 10.92 mg	SD = 22.60%	SD = 22.60 mg	SD = 2.55%	SD = 2.55 mg		
	Ambient (22 °C)	7	51.97%	51.97 mg	45.53%	45.53 mg	52.97%	52.97 mg	72.99%	72.99 mg	55.87%	55.87 mg
			SD = 6.06%	SD = 6.06 mg	SD = 16.50%	SD = 16.50 mg	SD = 14.70%	SD = 14.70 mg	SD = 9.69%	SD = 9.69 mg		
	Average rates		60.01%	60.01 mg	51.82%	51.82 mg	53.86%	53.86 mg	77.80%	77.80 mg	60.87%	60.87 mg
200 mg	Heated (80 °C)	7	45.75%	91.51 mg	52.17%	104.34 mg	54.33%	108.67 mg	37.59%	75.17 mg	47.46%	94.92 mg
			SD = 3.21%	SD = 6.41 mg	SD = 6.36%	SD = 12.73 mg	SD = 11.43%	SD = 22.86 mg	SD = 9.26%	SD = 18.52 mg		
	Ambient (22 °C)	7	28.93%	57.85 mg	26.16%	52.32 mg	26.19%	52.38 mg	28.28%	56.55 mg	27.39%	54.78 mg
			SD = 15.79%	SD = 31.59 mg	SD = 9.28%	SD = 18.57 mg	SD = 18.33%	SD = 36.67 mg	SD = 2.80%	SD = 5.60 mg		
	Average rates		37.34%	74.68 mg	39.17%	78.33 mg	40.26%	80.53 mg	32.94%	65.86 mg	37.42%	74.85 mg

*SD* Standard deviation

In the “room temperature water” condition, a statistically significant difference was found in favor of the “Wheel” filter compared to the cigarette filter (*p* < 0.01). No other significant difference was found between the other filters (*p* ≥ 0.09) for the water at room temperature. In the “heated water” condition, a statistically significant difference was found in favor of the “Wheel” filter when compared to the Sterifilt® (*p* < 0.01) and the cigarette filter (*p* = 0.03). No significant difference was found between the other filters (*p* ≥ 0.5) for heated water.

For the same filter, no significant difference was found between heated and room temperature water (*p* ≥ 0.3). The highest amounts of morphine found from the 100 mg capsules were from dissolutions made with heated water and then filtered through the “Wheel” filter (83 mg).

### Skenan® 200 mg capsules

Univariate analysis without interaction showed a significant difference in the amounts of morphine released into solution according to variations in filter and water temperature [*F* = 8.3; *p* < 0.01] when dissolving Skenan® 200 mg capsules.

The analysis with interactions found significant variations in extraction yields for variations in temperature of the dissolution water (*p* < 0.01), but not significant according to the filters used (*p* = 0.3), and there was no interaction between these variations in the preparation conditions (*p* = 0.1). The average extraction yields were 48% (i.e., 95 mg) of morphine per syringe in heated water and 27% (i.e., 55 mg) of morphine per syringe in water at room temperature (Table 1).

In the “room temperature water” condition, no significant difference was found between the filters (*p* ≥ 0.9). In the “heated water” condition, a statistically significant difference was found in favor of the the Sterifilt® compared to “Wheel” filter (*p* = 0.01). No significant difference was found between the other filters (*p* ≥ 0.09) for heated water.

For the same filter, the extraction yields were significantly different for the Sterifilt® (*p* ≤ 0.01) and the cigarette filter (*p* ≤ 0.01) between heated water and water at room temperature. This difference was not statistically significant for the “Wheel” filter (*p* = 0.7) and the Steribox® cotton (*p* = 0.1). The highest quantities of morphine found in the 200 mg capsules came from the dissolutions made with heated water, and then filtered through the “Sterifilt® Basic” filter (109 mg), even though there was no significant statistical difference between the filters (average rate: 95 mg).

## Discussion

The best extraction yields were obtained by dissolution in heated water, independently of the dosage of the Skenan® capsules (see Table 1). A significant effect of the filter used on the extraction yield was found only for the 100 mg capsules, in favor of the “Wheel” filter.

Regardless of variations in preparation conditions, the average extraction rates for the Skenan® 200 mg capsules were lower (water at 80 °C: 48%; water at 22 °C: 27%) than for the 100 mg capsules (water at 80 °C: 66%; water at 22 °C: 56%). In water at 22 °C, the extraction rates obtained were equivalent or slightly higher than the oral bioavailability of Skenan® 200 mg, while they were systematically higher for heated water and 100 mg capsules whatever the preparation conditions.

According to the *Merck Index*, 1 g of morphine sulfate dissolves in 0.7 ml of water at 80 °C, or 15.5 ml of water at 25 °C [27]. Thus, 1.6 and 3.1 ml of water at room temperature are required to dissolve 100 and 200 mg of morphine sulfate, respectively. The 2 ml of water used for our protocol, according to the recommendations of the PWIM, are thus insufficient to dissolve the totality of the active ingredient, thereby contributing to the low rates obtained for the 200 mg capsules.

This dissolution limit is associated with the phenomenon of filter saturation. The greater quantity of excipients and active principle contained in the 200 mg capsules saturates the solution more rapidly and obstructs the filters, increasing filtration difficulties. Direct heating of the solution improves the solubility of morphine, but increases the viscosity linked to the excipients [10, 25, 28]. Indeed, the hydrophilic matrix which constitutes the galenic of Skenan® microbeads gels in aqueous solution, a fortiori when heated, makes it more difficult to aspirate into the syringe. Heating the water separately before adding it to the Skenan® powder is an intermediate solution which favors the dissolution of the morphine, limiting the problems of filtration.

These elements also allow us to hypothesize why dissolving with heated water doubles the extraction rate of morphine for 200 mg capsules, while the effect is less pronounced for 100 mg capsules. The 200 mg capsules contain more morphine microbeads, and therefore more excipients, than the 100 mg capsules. This difference was unmistakable during the dissolution process. For the same volume of dissolution water, this higher quantity of excipients suspended in water may increase the difficulty to solubilize morphine, especially since its dissolution limits in cold water are exceeded for the 200 mg dose and the 2 ml volume. Heating the water compensates for this limitation, as the solubility of morphine increases with water temperature.

This problem of suspended particles is less important for 100 mg capsules and the dissolution limit of morphine is not reached for a dose of 100 mg and a volume of 2 ml. Heating the water still promotes dissolution, but to a lesser extent than the 200 mg capsule.

These problems of suspended particles and viscosity are added to those of filter access for PWIM and their possible fear that filtration will reduce the quantity of morphine in solution, which may sometimes lead them not to filter their solution, or the use of filters whose pores are the largest (cigarette filter and cotton of the Steribox®), at the risk of letting through insoluble or pathogenic elements [10, 28]. Our results show that this apprehension is unfounded. No significant difference in the quantity of morphine in solution was found according to the filter used for the 200 mg capsules. For the 100 mg capsules, the quantity of morphine found in solution was even greater when the “Wheel” filter was used than for the others.

The non-negative impact of filtration on the quantity of morphine in solution should encourage PWIM to favor the use of membrane filters that ensure that the clearest possible solution is obtained, thereby reducing the infectious and thrombotic problems associated with the injection of potentially pathogenic or insoluble particles [18–20, 22, 28, 29]. Studies that have evaluated the quality of suspended particulate matter filtration according to the filters used have found a superiority of filters marketed for this purpose (“Sterifilt® Basic” and “Wheel” filter) over cigarette or cotton filters [18–20].

The rigor of the manipulations and analyses carried out constitutes the main limitation of this study. It is necessary to emphasize that the dissolutions were carried out under the hygienic and working conditions of a pharmacological–toxicological analysis laboratory. These conditions inevitably differ from the real life of PWIM, of whom only a tiny minority benefit from the safety and hygiene conditions of a low-risk consumption room. The yields obtained in this study are only indicative, probably higher and less variable than those found in PWIM syringes in real conditions. In order to limit as much as possible the variations of the quantities of morphine in solution, we recommend that PWIMs ensure the best possible reproducibility of their dissolution protocol by avoiding any variation of the preparation conditions, and never have the preparation done by a third person. More broadly, the dissolution method reproduces the one described on the “Psychoactive” forum and seems to be popular among PWIM. However, there are probably other dissolution methods, unknown to us, which would bring a different result. There are also three more recent membrane filters, notably the “Sterifilt FAST®”, whose pores have the same diameter (10 µm) as those of the “Sterifilt® Basic”, but whose design allows for easier filtration, the “Sterifilt+®”, whose membrane has 0.22 µm pores, which also allows for the filtration of bacteria and fungi, and the “Universal wheel filter®” compatible with syringes with crimped needles.

Our results point out the limits of prescribing an oral formulation to persons who are known to inject it. It is now necessary to be able to assess the benefits that an injectable substitution could bring in terms of acceptability, efficacy, tolerance and even cost.

As the quantities of morphine actually injected are much lower than the unit dosage of the capsules, the doses could be easily reproduced using the vial of injectable morphine currently marketed for dosages ranging from 0.1 to 50 mg/ml. Costs could then be reduced, since a Skenan® 100 mg capsule is invoiced at about €1.50 per unit, whereas the average dose actually self-injected is about 70 mg. As a 500 mg/10 ml vial of morphine is invoiced at €5.67 per unit, the administration of 70 mg of injectable morphine, i.e., 1.4 ml, would cost €0.80. The large variety of available dosages of injectable morphine would make it possible to adapt the prescribed quantity to the real needs of PWIM.

This latter would have a better control of the reproducibility of self-administered doses, reducing the risks of involuntary intoxication.

Injectable substitution with morphine would also reduce the infectious and thrombotic risks to which PWIM are exposed. Experimentation with such an intravenous substitution could be carried out within the framework of the opening of supervised injection rooms “Haltes Soins Addictions” (“Drop-in Addiction Care”), prolonging the French experimentation with lower risk consumption rooms [30, 31], after a rigorous evaluation of its benefits and risks within a clinical trial. These allow supervised self-administration of substances, particularly morphine from illicit markets, the drug most injected daily (24%) in these consumption rooms [32], while conforming to the principles of asepsis and promoting the entry of PWIM into multidisciplinary care which can be beneficial to them [30, 31]. This care setting would be suitable for the eventual provision of an injectable substitution, alone or in addition to a validated OST, whose effectiveness has been scientifically documented [33–35]. In various countries (including Switzerland, Netherlands, Germany, Denmark, and Canada), the legal prescription of diacetylmorphine- or hydromorphone-assisted treatment under strict supervision has indeed proven to be effective and well tolerated [33, 36–40]. Supervised injection rooms also make it possible to set up social care for the most precarious PWIM, provide support and education on RHR related to injection, and even initiate cognitive-behavioral therapy for those subject to behavioral dependence on injection. The PWIM concerned could then benefit from progressive management, with an injectable substitution, transiently concomitant to the oral form. In line with an approach aimed at risk reduction, and in accordance with the most recent recommendations on the issue [41], these prescriptions would be accompanied by the delivery of a naloxone kit for the emergency treatment of opioid overdose and RHR equipment specific to the needs of these PWIM, such as disinfectant, equipment adapted to their individual needs (large volume syringe, different needles, etc.) and a recycling circuit, which is necessary for used equipment.

## Conclusions

No procedure for dissolving Skenan® led to the complete dissolution of the morphine it contains. The best extraction yields were obtained by dissolution in previously heated water, independently of the dosage of the Skenan® capsules. RHR membrane filters never negatively impact morphine extraction compared to “cotton” or cigarette filters. Offering an injectable substitution to persons who inject morphine would make it possible to reduce the risks and damage, particularly overdoses, as well as variations in dosage due to preparation methods.

## Supplementary Information

**Additional file 1. Figure S1.** Risk reduction material used for the dissolution of Skenan® capsules. **Figure S2.** Dissolution protocol of Skenan® capsules.

## Data Availability

The dataset supporting the conclusions of this article is hosted by Mendeley web platform and freely available: https://data.mendeley.com/datasets/7kry5bdp68/1.

## Abbreviations

OST: Opioid substitution treatments
OUD: Opioid use disorder
PWIM: People who inject morphine intravenously
RHR: Risk and harm reduction
