# Supplementary material for: Intravenous misuse of slow-release oral morphine capsules: how much morphine is injected?
Source: Harm Reduct J. 2023 Apr 27;20:59. doi: 10.1186/s12954-023-00781-2 (PMC10134660; doi:10.1186/s12954-023-00781-2)
Supplement: Supplementary file 1 — Additional file 1. Figure S1. Risk reduction material used for the dissolution of Skenan® capsules. Figure S2. Dissolution protocol of Skenan® capsules. [file 12954_2023_781_MOESM1_ESM.docx]

**Intravenous misuse of slow-release oral morphine capsules: how much morphine is injected?**

Célian Bertin* ^1,2,3^, Edouard Montigne^1^, Sarah Teixeira^1^, Florent Ferrer^1^, Louis Lauwerie^1^, Damien Richard^1^, Nicolas Authier^1,2,3^

^1^ Université Clermont Auvergne, CHU Clermont-Ferrand, Inserm 1107 Neuro-Dol, Service de Pharmacologie Médicale, Centres Addictovigilance et Pharmacovigilance, Centre Evaluation et Traitement de la Douleur, F-63003, Clermont-Ferrand, France.

^2^ Observatoire Français des Médicaments Antalgiques (OFMA) / French Monitoring Centre for Analgesic Drugs, CHU Clermont-Ferrand, Université Clermont Auvergne, F-63001, Clermont-Ferrand, France.

^3^ Fondation Institut Analgesia, Faculté de Médecine, F-63001, Clermont-Ferrand, France.

*Corresponding Author

Célian Bertin, Service de Pharmacologie Médicale, BP-69, CHU Gabriel Montpied, 58 Rue Montalembert, 63000 Clermont-Ferrand, France

Fax: +33,473,751,823
Tel: +33,473,751,822

Email address: [cbertin@chu-clermontferrand.fr](mailto:cbertin@chu-clermontferrand.fr)

**Figure S1.** Risk reduction material used for the dissolution of Skenan® capsules.
From left to right: sterile cup (Stericup®), sterile syringe (2 ml), sterile water for injection (5 ml single-dose), sterile "Sterifilt® Basic" filter, cigarette filter, sterile cotton filter from the "Steribox®" risk reduction kit, 25 mm "Spinning top" filter.


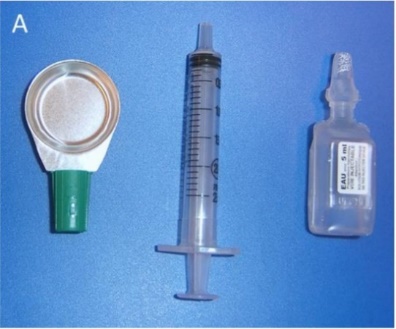

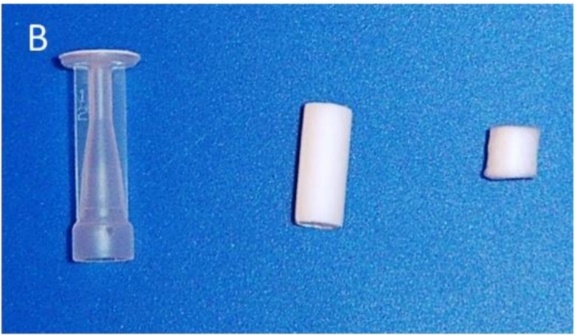

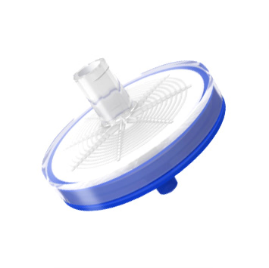

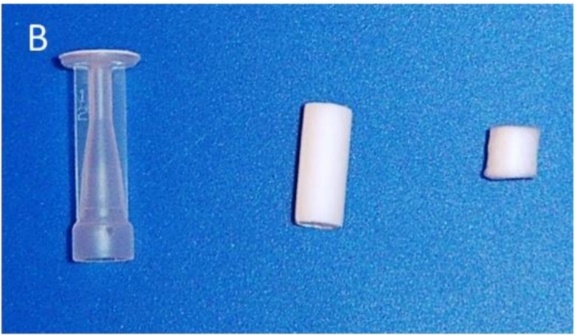

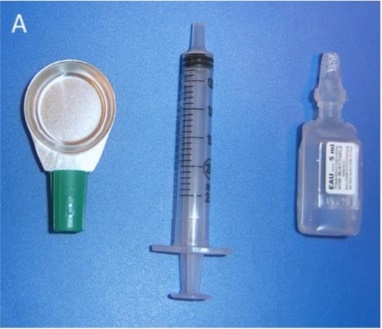

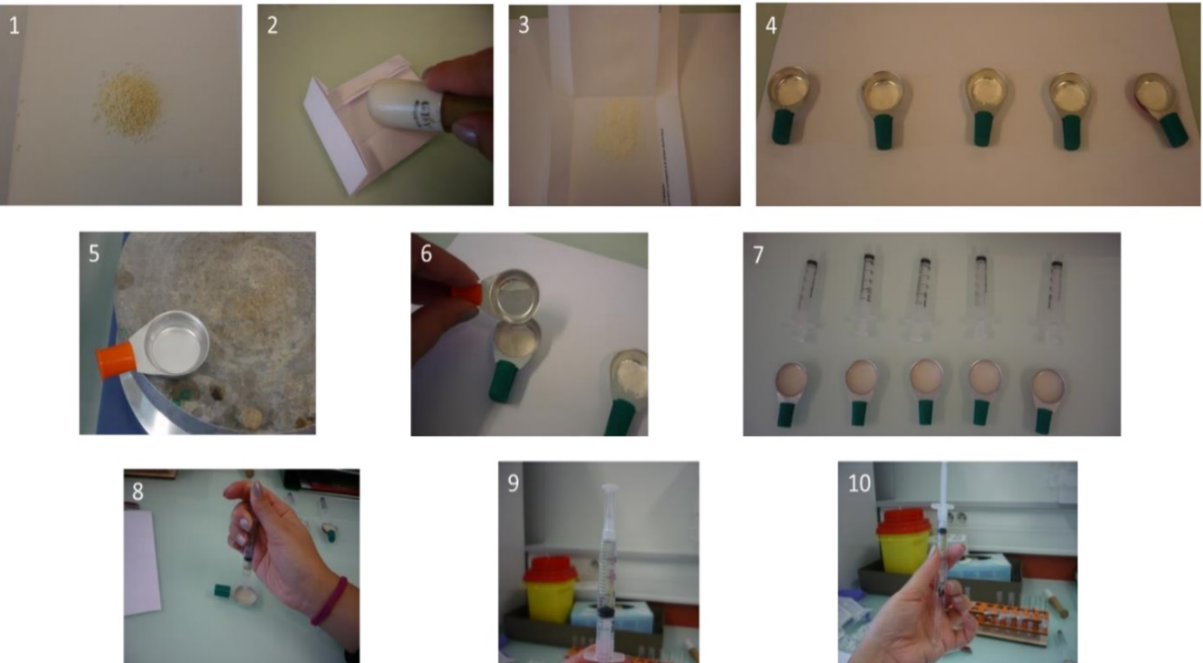

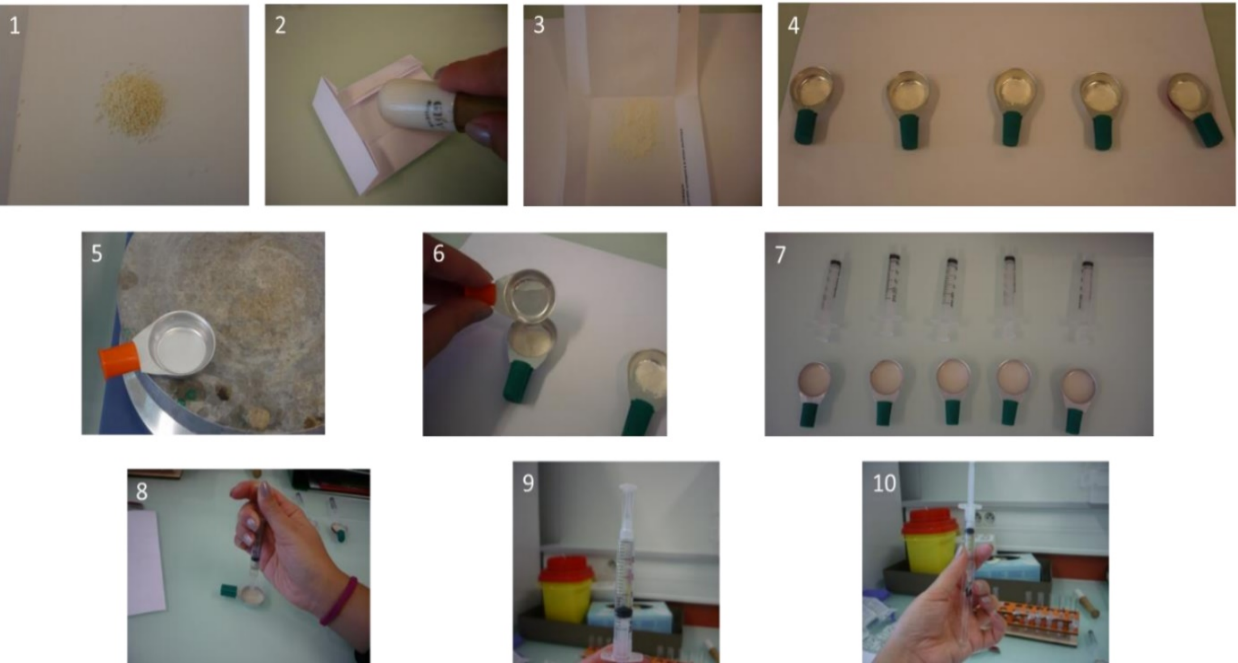

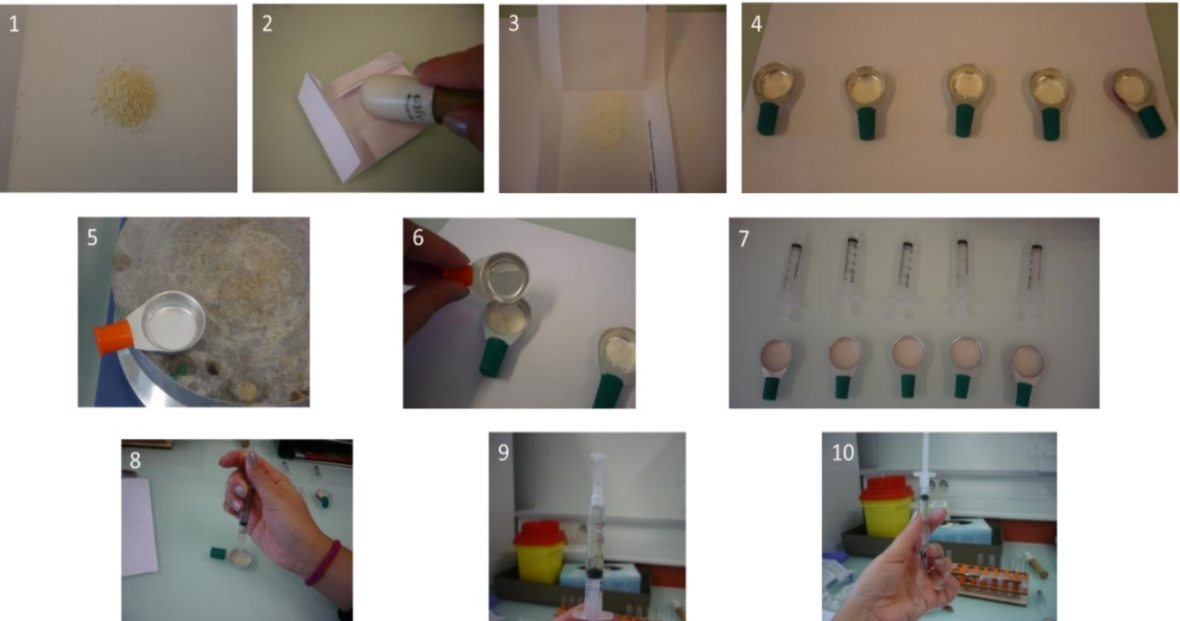

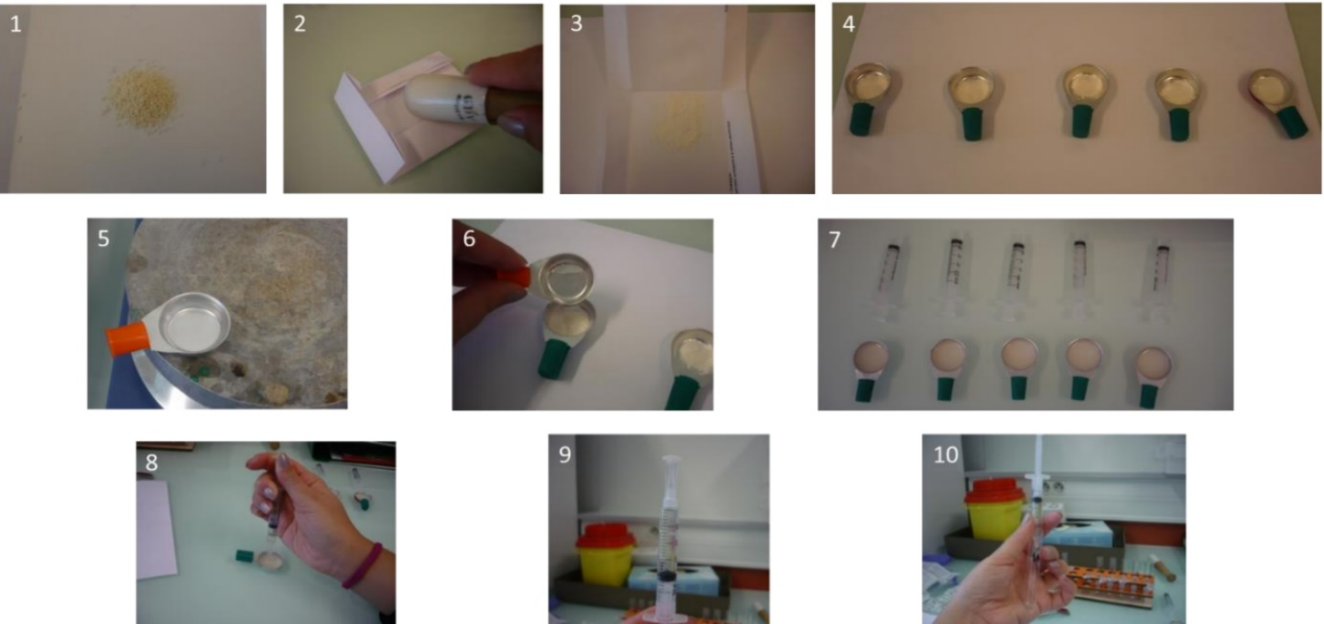

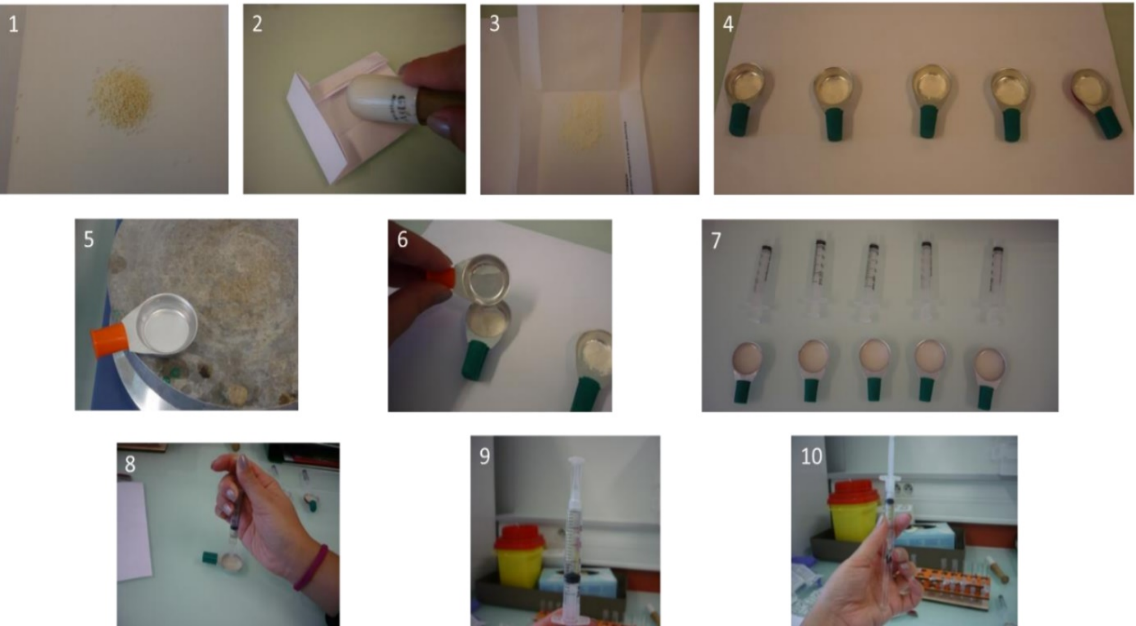

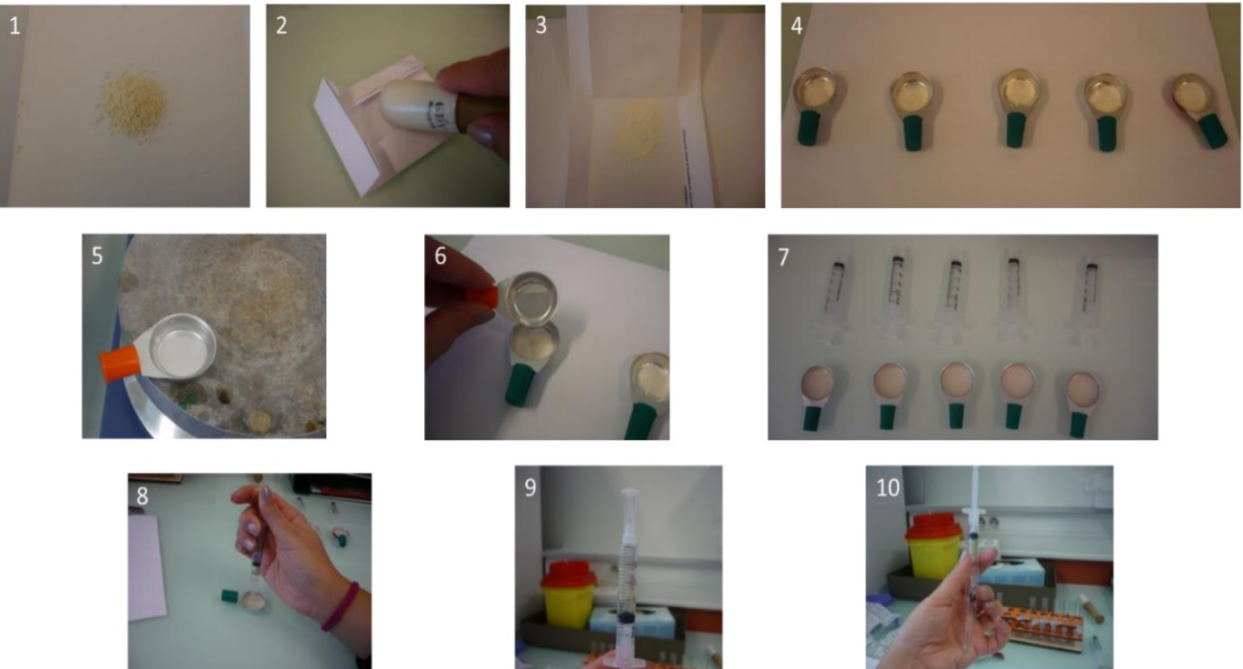

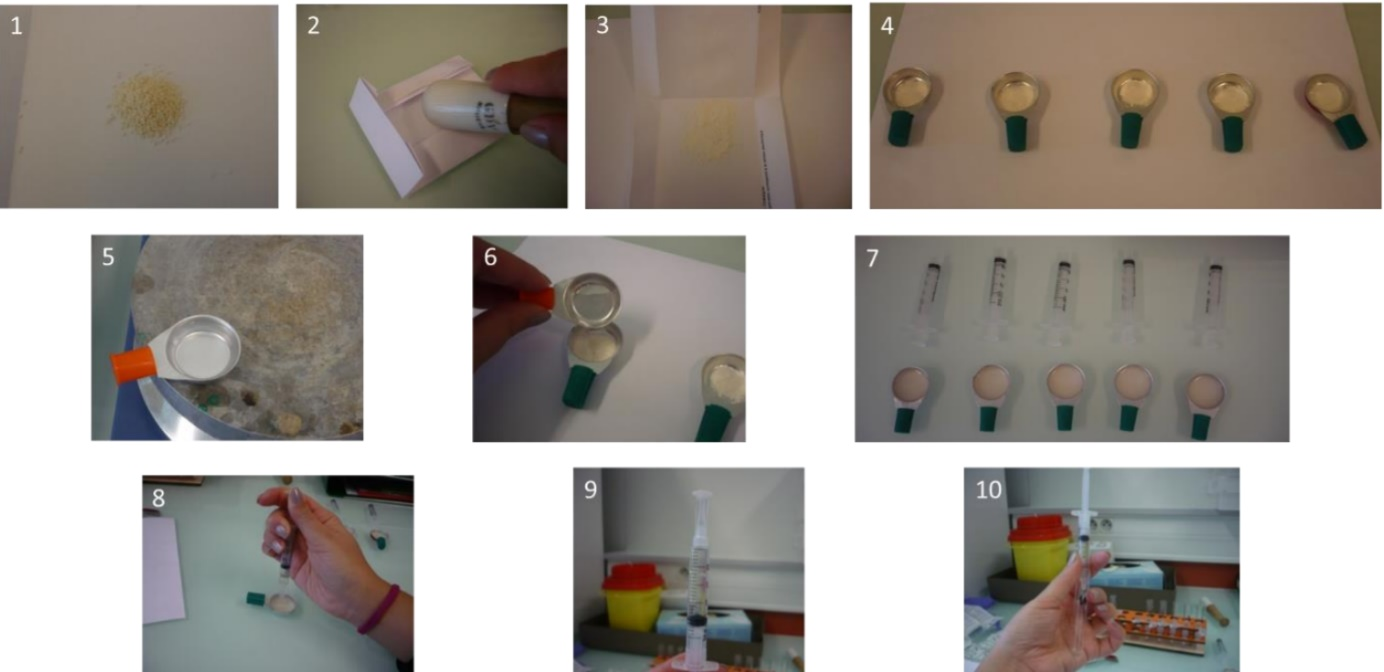

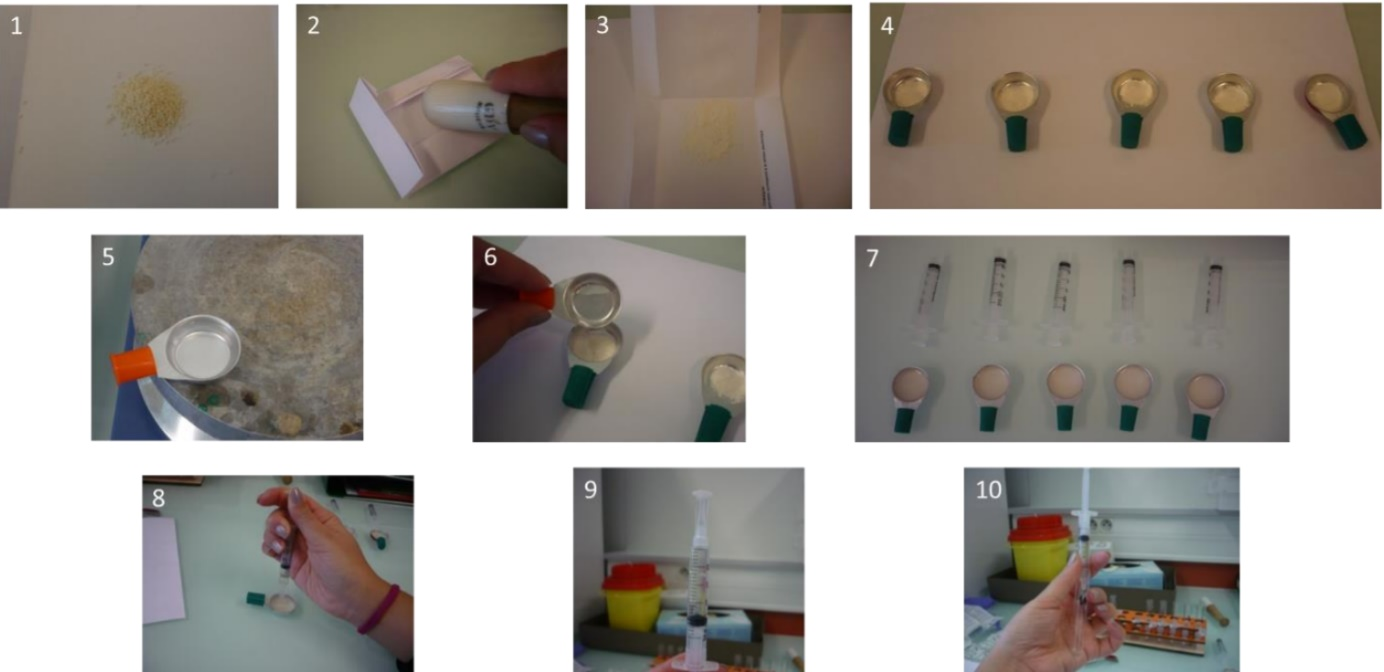

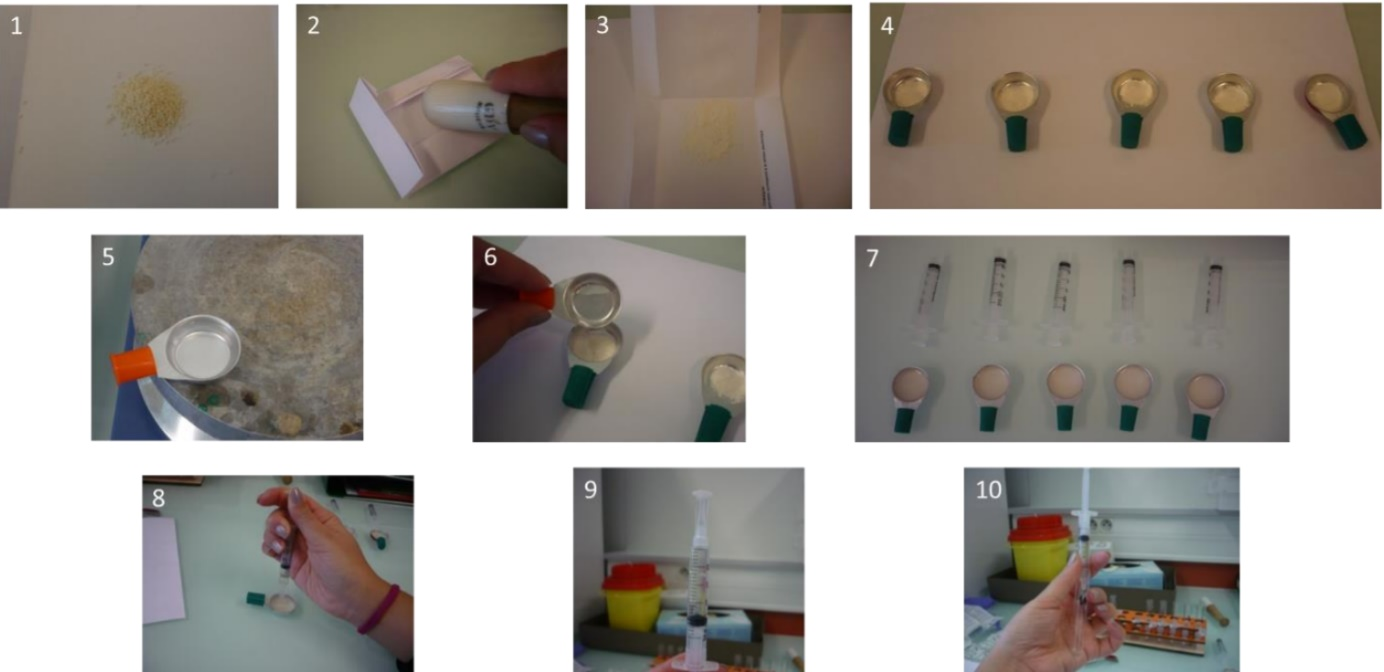

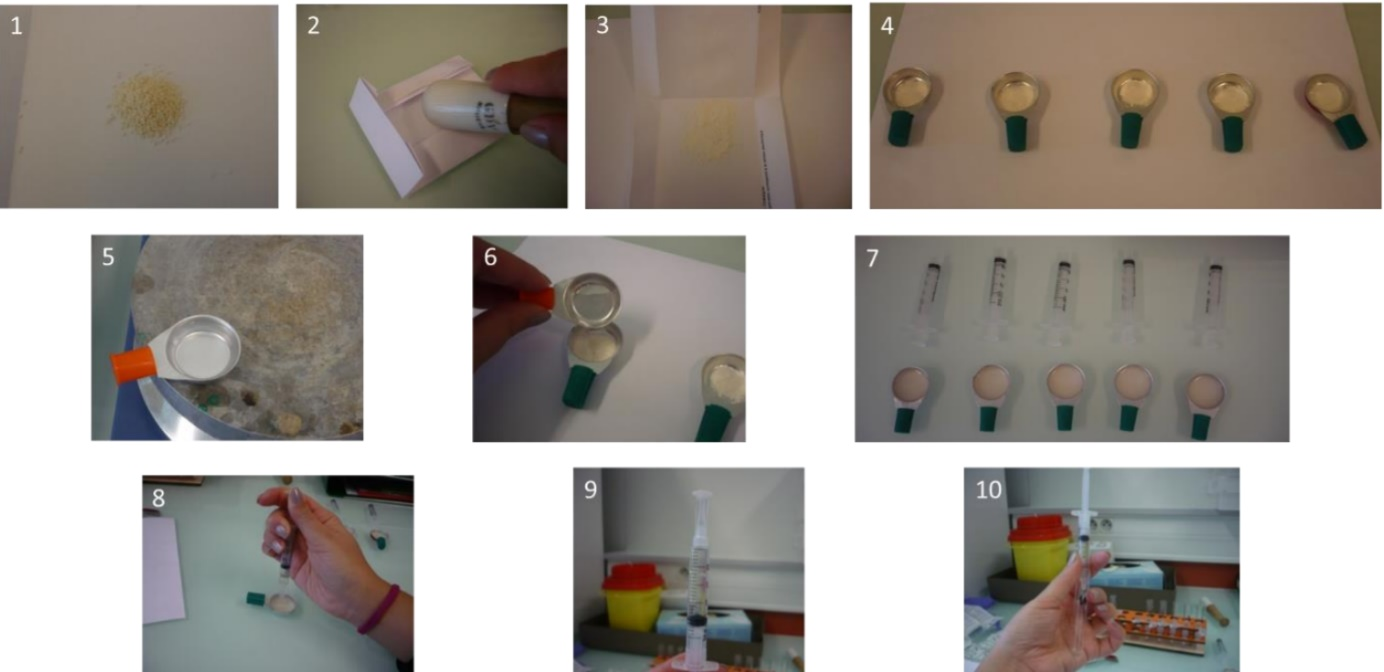


**Figure S2.** Dissolution protocol of Skenan^®^ capsules.

From left to right, and from top to bottom: reduction of the microbeads to powder (steps 1 to 4), dissolution in sterile water previously heated (steps 5) or at room temperature (steps 6 and 7), then filtration using the "Sterifilt®" in this figure (steps 8 and 9), before transfer to a hemolysis tube (step 10) for spectrophotometric analysis.
